# Effect of Film-Forming Alginate/Chitosan Polyelectrolyte Complex on the Storage Quality of Pork

**DOI:** 10.3390/molecules22010098

**Published:** 2017-01-06

**Authors:** Dominika Kulig, Anna Zimoch-Korzycka, Żaneta Król, Maciej Oziembłowski, Andrzej Jarmoluk

**Affiliations:** Department of Animal Products Technology and Quality Management, Wroclaw University of Environmental and Life Sciences, Chelmonskiego Street 37, 51-630 Wroclaw, Poland; anna.zimoch@up.wroc.pl (A.Z.-K.); zaneta.krol@wp.pl (Ż.K.); maciej.oziemblowski@up.wroc.pl (M.O.); andrzej.jarmoluk@up.wroc.pl (A.J.)

**Keywords:** meat quality, chitosan, alginate, polyelectrolyte complex, coating, antimicrobial, antioxidant properties

## Abstract

Meat is one of the most challenging food products in the context of maintaining quality and safety. The aim of this work was to improve the quality of raw/cooked meat by coating it with sodium alginate (A), chitosan (C), and sodium alginate-chitosan polyelectrolyte complex (PEC) hydrosols. Antioxidant properties of A, C, and PEC hydrosols were determined. Subsequently, total antioxidant capacity (TAC), sensory quality of raw/cooked pork coated with experimental hydrosols, and antimicrobial efficiency of those hydrosols on the surface microbiota were analysed. Application analyses of hydrosol were performed during 0, 7, and 14 days of refrigerated storage in MAP (modified atmosphere packaging). Ferric reducing antioxidant power (FRAP) and (2,2-diphenyll-picrylhydrazyl (DPPH) analysis confirmed the antioxidant properties of A, C, and PEC. Sample C (1.0%) was characterized by the highest DPPH value (174.67 μM Trolox/mL) of all variants. PEC samples consisted of A 0.3%/C 1.0% and A 0.6%/C 1.0% were characterized by the greatest FRAP value (~7.21 μM Fe^2+^/mL) of all variants. TAC losses caused by thermal treatment of meat were reduced by 45% by coating meat with experimental hydrosols. Application of PEC on the meat surface resulted in reducing the total number of micro-organisms, psychrotrophs, and lactic acid bacteria by about 61%, and yeast and molds by about 45% compared to control after a two-week storage.

## 1. Introduction

The main cause of sensory, functional, and nutritional quality deterioration in meat during storage are microbial spoilage and lipid/protein oxidation [1]. Metabolic and other processes lead to the formation of reactive oxidative species (hydroxyl, superoxide, peroxide, and nitric oxide radicals) that are able to interact with proteins and lipids during meat aging and storage [2]. Lipid oxidation results in unpleasant taste and odour, colour deteriorations, protein degradation, and the accumulation of toxic compounds, which could affect consumers’ health [2,3]. The oxidation of meat proteins causes the development of carbonyls, hydroperoxidases, and sulfoxides, deterioration of texture, and loss in water-holding capacity. The specific chemical composition of meat promotes microbial growth, which, over time, may lead to deterioration of meat quality or/and its spoilage. When unacceptable levels of microorganisms are present in raw meat, off-odours, off-flavours, discoloration and slime develop and meat becomes unappealing and unsuitable for human consumption [4,5]. Qualitative and quantitative changes in meat reduce the length of its shelf-life. Thus, to maintain the quality of meat, the oxidation process and microbial growth should be limited to a minimum. According to Leister, the application of hurdle technology seems to be the most effective method of improving shelf-life, safety and quality of meat and meat products [6]. Hurdle technology refers to a combination of multiple technologies, which, used simultaneously, may provide safe quality levels throughout the distribution channel [7]. In this work the combination of low temperature storage, polyelectrolyte coating, and modified atmosphere packaging (MAP)-packaging were applied. Application of active coatings fits into the principles of hurdle technology and could be easily introduced to industrial food production as an additional production step. Ojagh et al. developed chitosan coatings enriched with cinnamon oil to increase the shelf-life of cold-stored trout fillets [8]. Jeon et al. discovered that chitosan coatings were effective in protecting lipids from oxidation when applied to herring and cod fillets [9]. Suman et al. showed that coating ground beef patties with chitosan reduced TBARS (thiobarbituric acid reactive substances) values and improved the surface red colour of patties compared to non-coated samples [10]. Zimoch-Korzycka et al. presented that HPMC (hydroxypropyl methyl cellulose)-chitosan films were suitable for meat and meat products due to their desired thermo-mechanical, barrier, and antimicrobial properties [11]. The study of Solutos et al. showed that chitosan can be used to extend the shelf-life of fresh pork sausages due to its antimicrobial and antioxidant properties [12]. As reported by Wanstedt et al. alginate coatings have been used to retard the development of oxidative off-flavours in pre-cooked meat patties [13]. The coating with 2% sodium alginate with preservatives improved the overall appearance, colour, juiciness, flavour, texture, and overall palatability of buffalo meat patties [14]. 

Lower meat quality is caused mainly by microbial spoilage and lipid/protein oxidation, therefore applied coatings should act bidirectionally: reducing the rate of microbial growth and inhibiting the oxidation process. Coatings should also possess optimum physicochemical properties to be applied on food surfaces. In earlier studies, we confirmed the desirability of connecting alginate and chitosan due to the improvement of their thermal and mechanical properties, as well as chemical stability [15]. The sodium alginate-chitosan polyelectrolyte complex is formed by the interaction between the dissociated functional groups: an anionic carboxyl group of alginate and cationic amino group of chitosan. Due to the use of oppositely-charged polymers and the application of optimized reaction conditions (pH, temperature, polymers ratio, order and speed of mixing, chemical characteristic of used polymers, etc.) polyelectrolyte complexation precludes the need for additional chemical cross-linkers and results in the formation of hydrogel structure. Subsequently, the obtained hydrogel can be applied directly to the products surface. 

The aim of this study was to evaluate how sodium alginate, chitosan, and sodium alginate-chitosan polyelectrolyte complex hydrosols influenced meat quality during 0, 7, and 14 days of refrigerated storage in MAP. At first, we determined the antioxidant properties of hydrosols and then the total antioxidant capacity and sensory quality of raw and cooked meat coated with those experimental hydrosols. Additionally, antimicrobial efficiency of A, C, and PEC hydrosols on the surface microbiota of raw meat was analysed. It was proven that sodium alginate-chitosan complex physicochemical features are strongly dependent on the polymer ratio/binding degree [15]. However, the effect of polyelectrolyte complexation on the biological activities of sodium alginate-chitosan complex and food application experiments was not earlier described in the literature, therefore, it became the subject of this study. Better understanding of the dynamic properties of this system will help to introduce it to the food market in the future.

## 2. Results and Discussion

### 2.1. First Part of Experiment—Analysis of Hydrosols

#### Free Radical Scavenging Activity of DPPH (2,2-Diphenyll-picrylhydrazyl) and Ferric Reducing Antioxidant Power (FRAP) of Experimental Hydrosols

**Figure 1:**
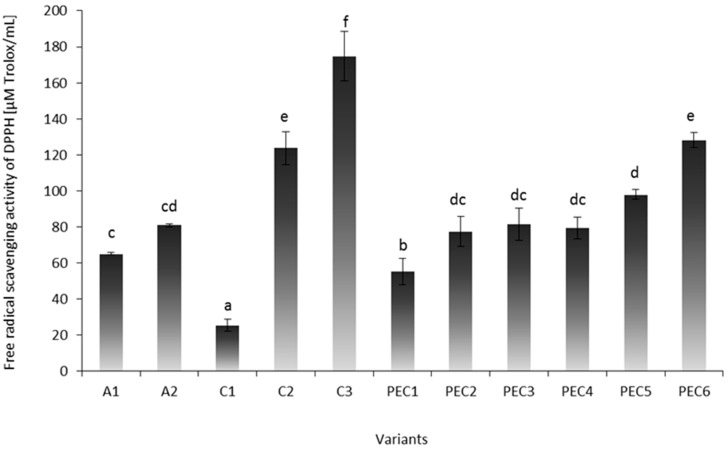
The antioxidant activity of sodium alginate (A1, A2), chitosan (C1, C2, C3), and polyelectrolyte complex (PEC1-PEC6) hydrosols determined by free radical scavenging of DPPH. Values with different letters (a–f) differ significantly (*p* < 0.05), *n* = 3.

One important mechanism of antioxidation involves the scavenging of hydrogen radicals. Free radical scavenging activities of DPPH of experimental hydrosols are presented in Figure 1. Single A hydrosols were characterized by lower DPPH values (~73.04 μM Trolox/mL) compared to C samples (~107.98 μM Trolox/mL), which is in accordance with the study of Zimoch-Korzycka et al. [16]. DPPH radical scavenging activity of C increased in direct proportion to its concentration (*p* < 0.05). Sample C3 was characterized by the highest DPPH value (174.67 ± 13.70 μM Trolox/mL) of all variants. Study of Zimoch-Korzycka et al., Trung et al., and Muzzarelli et al. confirmed that chitosan in higher concentrations has greater scavenging abilities [16,17,18]. Antioxidant activity of chitosan has been mainly attributed to free radical scavenging activity of its hydroxyl and amine groups. Xia et al. [19] suggested that free radicals can react with the hydrogen ion from NH_3_+ to form a stable molecule. Xue et al. [20] indicated that the scavenging activities of chitosan derivatives against hydroxyl radical was derived from: (1) hydrogen atom abstraction reaction between the hydroxyl groups in the polysaccharide unit and the hydroxyl radical; (2) the residual free amino groups that can react with the hydroxyl radical; or (3) the NH_2_ groups can form NH_3_+ then reacting with the hydroxyl radical through addition reaction.

Complexation of chitosan with sodium alginate resulted in minor deterioration of the antioxidant capacity of PEC hydrosols. The greatest values of mixed samples were obtained for samples PEC6 (128.13 ± 4.24 μM Trolox/mL) and PEC5 (97.93 ± 2.73 μM Trolox/mL), with the highest doses of both polymers and a 0.60–0.80 polymer ratio. DPPH values of samples PEC2 and PEC3 with the lowest polymer ratio (R = 0.30–0.40) were 80.44 ± 7.57 μM Trolox/mL (*p* > 0.05). Despite the equal polymer ratio of samples PEC1 and PEC6 (R = 0.60), sample PEC1 was characterized by the most unfavourable scavenging properties. The above results confirmed that the concentration of polymers in PEC has a greater effect on scavenging abilities than the polymer ratio (a higher polymer ratio results in a lower number of free reactive amino groups in the chitosan chain). On the other hand, Lin and Chou [21] noted that disaccharide chitosan derivatives with low disaccharide substitution (DS) (20%–30%) exhibited higher DPPH radicals, superoxide anion radicals, and H_2_O_2_-scavenging activity than those with higher DS (60%–70%). According to the authors, the more free amino groups present (low DS) in chitosan derivatives, the higher the scavenging effect toward DPPH radicals and superoxide anion radicals. After formation of PEC, the number of un-bonded NH_3_+ groups (able to interact with radical/metal ion) presented in chitosan is lowered by the interaction with carboxylic groups of alginate, which should be observed as the deterioration of antioxidant properties. On the other hand both used biopolymers possess some biological activities and an interactional effect of chitosan and sodium alginate was observed in some samples (PEC3, PEC6).

**Figure 2:**
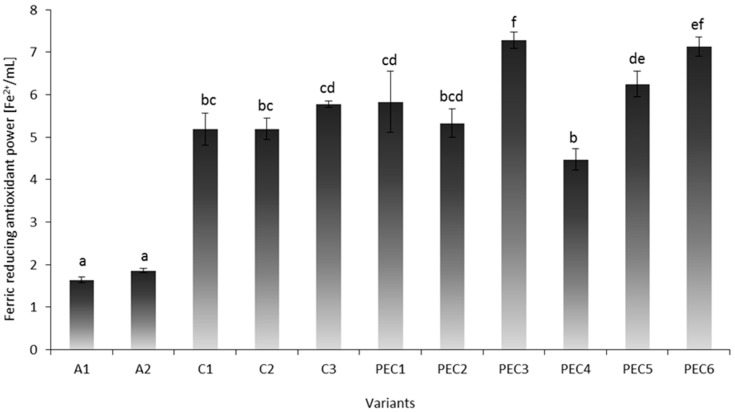
The antioxidant activity of sodium alginate (A1, A2), chitosan (C1, C2, C3), and polyelectrolyte complex (PEC1–PEC6) hydrosols determined by ferric reducing antioxidant power (FRAP). Values with different letters (a–f) differ significantly (*p* < 0.05), *n* = 3.

Transition metal ions can initiate lipid peroxidation and start a chain reaction, which leads to deterioration of meat quality attributes. In the present study, FRAP analysis was performed to investigate the chelating ability of sodium alginate, chitosan and PECs with various polymer ratios. Figure 2 shows results of FRAP analysis. Ferric reducing antioxidant power of A1–A2 samples was three times lower (~1.75 μM Fe^2+^/mL) than C1–C3 hydrosols (~5.39 μM Fe^2+^/mL). The effect of sodium alginate concentration was not observed. The increase in the chitosan dose improved scavenging ability of PEC samples, which may be observed in PEC3 and PEC6 samples. Those samples were characterized by the highest dose of chitosan in composition and, thus, the greatest ferric reducing antioxidant power (~7.21 μM Fe^2+^/mL) of all variants. Sample PEC4 was characterized by the lowest FRAP value of all complexed samples. The aforementioned sample was characterized by the excess of alginate in composition, which could inhibit antioxidant properties of chitosan. Chitosan is able to form complexes with the transition metals and some metals from groups 3–7 of the periodic table. The heavy metal-chitosan complexes are believed to form as a result of dative bonding. This involves the donation of nonbonding pairs of electrons from the nitrogen, and/or the oxygen of the hydroxyl groups, to a heavy metal ion [22]. According to Kamil et al. [22] chelation ability of chitosan depends on its molecular weight and degree of polymerization of the glucosamine unit.

Non-conformity of DPPH and FRAP results stems from a different mechanism of reaction and various binding affinity of free radical/metal ion by used biopolymers. Scavenging of DPPH free radicals allows the evaluation of the hydrogen-donating potency of antioxidative substances when FRAP assay reflects the reductive antioxidant power of ferric-tripyridyltriazine (Fe^III^-TPTZ) complex to its ferrous (Fe^II^) form. Lin and Chou [21] mentioned that, unlike the effects observed on the scavenging effect for DPPH and H_2_O_2_, no regular trend concerning the effect of disaccharide substitution on the Cu^2+^-scavenging activity of the disaccharide chitosan derivatives can be drawn, which is in accordance with the results obtained in the present work.

### 2.2. Second Part of Experiment—Application Analysis

#### 2.2.1. Total Antioxidant Capacity (TAC) Measurements

The total antioxidant capacity (TAC) of pork meat (*longissimus thoracis*) was measured to evaluate changes in TAC by storage time, thermal treatment, and application of polyelectrolyte complex hydrosol on the meat surface. The QUENCHER procedure is a method of TAC measurement of solid foods, based on the interaction occurring at the interface between the solid matrix and a liquid, coloured radical probe [23]. The effect of storage time and hydrosol composition on TAC values of raw/cooked meat were presented in Table 1.

Covering raw meat with A2, C3, and PEC6 turned out to be the most effective method of increasing TAC values in DPPH assay. Similarly, application of samples A2 and PEC6 on the meat surface resulted in the highest values of TAC in FRAP assay. As a mean, these samples were characterized by 12% higher DPPH and up to 35% higher FRAP values than control meat samples not covered with experimental hydrosols (*p* < 0.05). Similar results were obtained for samples subjected to heat treatment, except that the average values of DPPH were slightly lower (3%), while in the FRAP method an increase of ferric reducing antioxidant power by about 50% compared to control group was observed (*p* < 0.05). According to the above, more radical differences in total antioxidant capacity of meat were obtained in FRAP analysis. Each variant of hydrosols had a positive impact on the FRAP value of raw and cooked meat compared to the control. Thermal process significantly affected the decrease in TAC values of meat control and meat-hydrosol samples. Serpen et al. observed a rapid increase of FRAP values in beef, pork, chicken, and fish due to heating (180 °C) of samples in the oven [24]. The authors explained such a direction of changes by accumulation of Maillard reaction products (MRPs). In this work the Maillard reaction was inhibited by high water activity (cooking in water) and mild thermal treatment conditions (80 °C). Additionally, direct contact with water could cause the release of antioxidant substances from meat to an aqueous medium. Nevertheless, coating of meat with experimental hydrosols reduced the loss in total antioxidant capacity caused by thermal treatment of meat by 45%. Hydrosol hinders the contact of the meat surface with the external environment, thus inhibiting the negative influence of cooking on meat tissue. Applied polymers are stable in the temperatures up to 160–180 °C (depending on sodium alginate/chitosan ratio) which may be the reason of their protective effect [15]. Barrier properties of polymers may also be linked with their rheological properties. Our earlier studies confirmed that complexes with the highest dose of polymers and the greatest binding degree (R) were characterized by the greatest viscosity and consistency index. Additionally, good wettability and the gel-like structure of PEC were confirmed, which enhanced adhesion of the complex to the product [25].

The protective effect of hydrosols on raw and cooked meat is also linked with biological activities of used polymers. Active hydroxyl and amino groups present in those polysaccharides act as metal-chelating agents [26]; for example, chitosan has the ability to chelate free iron, released by myoglobin degradation during post-harvest meat storage and thermal processing [22]. Other studies also confirmed antioxidant properties of chitosan. Suman et al. reported that coating ground beef patties with chitosan reduced TBARS values when compared to non-coated samples [10]. The study of Solutos et al. showed that chitosan can be used to extend the shelf-life of fresh pork sausages due to its antimicrobial and antioxidant properties [12].

DPPH and FRAP values of raw/cooked meat-hydrosol samples increased in direct proportion to the storage time. In the case of the control sample, the opposite direction of changes was noted, the value of total antioxidant capacity of meat gradually decreased or fluctuated during two weeks of storage. The rate of lipid/protein oxidation depends on the balance between factors which promote oxidation: (1) endogenous pro-oxidant substances (e.g., haemoglobin and other iron porphyrins); (2) composition of substrates prone to oxidation: fatty acids, cholesterol, proteins, and pigments; and (3) the amount of available oxygen; and those that counteract this process like: (1) endogenous non-enzymatic antioxidants: vitamin E, vitamin C, carotenoids, ubiquinols, polyphenols, and cellular thiols; (2) enzymes (superoxide dismutase, catalase, and glutathione peroxidase); and (3) the presence of exogenous reducing agents [24,27]. Polyelectrolyte hydrosols may impede the operation of native pro-oxidants contained in the meat and from external sources and have a protective effect on natural antioxidants contained in the meat tissue, thereby increasing the reducing power of meat during storage.

#### 2.2.2. Microbiological Analysis

Total number of micro-organisms (TNM), yeast and molds (YM), psychrotrophs (P), lactic acid bacteria (LAB) and Enterobacteriaceae (E) of raw meat coated with A, C, PEC, and LA, after 0, 7, and 14 days of storage at 4 °C in MAP atmosphere are presented in Table 2. Control meat samples were found to have an average 41% higher population of TNM, YM, P, and LAB compared to coated meat. Enterobacteriaceae are considered as general indicators of the hygienic state of fresh and processed meat. Presented analysis confirmed their absence on the meat surface, which indicates good production practices and hygienic conditions of the meat plant. The microbial growth increased gradually after 0, 7, and 14 days of storage. At zero days of storage, TNM of the control meat sample was 3.80 CFU/cm^2^ and met the microbiological requirements of European Union standards–acceptable at <4.00 CFU/cm^2^ [28]. After 7 and 14 days of storage TNM of meat control exceeded EU microbiological criteria for carcasses of pigs (unacceptable > 5.0 log CFU/cm^2^) and were 6 and 7 log CFU/cm^2^, respectively. According to the above results, even application of controlled atmosphere conditions (MAP packaging) and chill temperature storage (4 °C) were not sufficient to inhibit the undesirable microbiological changes in the raw meat material. Carbon dioxide inhibits the multiplication of *Pseudomonas* spp., but lactic acid bacteria may still become predominant. Lactic acid bacteria are identified as spoilage bacteria of sausages and processed meat and their growth affect microbial quality of vacuum and MAP-packed raw meat [29]. In the present work, the inhibited growth of LAB at 0 day of storage was observed and LAB colonization on meat surfaces increased after seven days of storage and continued to 14 days due to limited oxygen content. When compared to control samples, meat covered with C, PEC, and LA solution had almost 50% lower survival rates of TNM, YM, P, and LAB. Microbial colonization of those samples surface was slower than in control samples.

The presented research confirmed the antimicrobial effects of chitosan on meat microbiota. Microbial colonization decreases with increasing concentration of chitosan, which was also confirmed by Ulbin-Figlewicz et al. and Zimoch-Korzycka and Jarmoluk [30,31]. Three models of antibacterial action of chitosan have been proposed so far: (1) interaction between positively-charged chitosan molecules and negatively-charged microbial cell membranes; (2) binding of chitosan with microbial DNA; and (3) chelation of metals, suppression of spore elements and binding to essential nutrients to microbial growth [32]. The exact mechanism is not fully understood and several other factors may contribute to the antibacterial action, but it seems that the most acceptable model is the electrostatic interaction between the protonated NH_3_+ groups and the negative microbial residues [33]. Since chitosan hydrosol has to be prepared by dissolution of powdered chitosan in lactic acid solution, the effect of this acid on antimicrobial properties must be considered. Significantly greater antimicrobial properties (TNM, YM, P) of 0.5% LA solution were observed at the initial time of storage (2.11 log CFU/cm^2^) compared to 0.5%–1.0% chitosan solutions (2.57 log CFU/cm^2^). After 7 and 14 days of storage 1.0% solution of chitosan was characterized by greater ability to reduce total viable counts of meat surface (3.49 log CFU/cm^2^) than lactic acid solution (4.29 log CFU/cm^2^) which confirms its antimicrobial properties (*p* < 0.05). In addition, all doses of C had a better effect on YM reduction than lactic acid solution. After 7–14 days of storage the reduction ability of psychrotrophs by 1.0% of chitosan solution was similar (*p* > 0.05) to lactic acid solution. Chitosan action was significantly more effective in LAB reduction than lactic acid solution throughout the entire storage period, with average log CFU/cm^2^ of 2.25 and 3.18, respectively. In meat covered with sodium alginate hydrosol (0.3%, 0.6%) mortality of surface microbiota was slightly reduced compared to control (about 9% of all tested groups of microorganisms). Similar results were obtained by Song et al. who observed a reduction of TNM in samples of bream coated with 1.5% sodium alginate hydrosol compared to control sample. Authors assumed that the coating acts as a barrier against oxygen transfer and leads to inhibition of growth of the aerobic bacteria [34].

Interestingly, after coating meat with PEC, the greatest reduction of surface meat microbiota (TNM, YM, P, LAB) was observed. An average number of tested microbiota during whole storage period of samples PEC1–PEC6 was 2.05 log CFU/cm^2^. PEC has nearly 30% greater reduction ability of the number of surface microorganisms than pure chitosan. Other authors obtained similar results of microbiological analysis of polyelectrolyte complexes. Yang et al. reported that the inhibition of *Pseudomonas aeruginosa* in polypropylene fabrics impregnated with chitosan at different concentrations increased with an increasing polypropylene/chitosan ratio. The authors explained that since chitosan is a cationic polysaccharide, the charge density of the fabric containing it increased and improved the absorption of cells which were negatively charged, thereby decreasing the viable cell count [35]. Ortega-Ortiz et al. noted that antibacterial activity of poly(acrylic acid) (PAA)-chitosan interpolyelectrolyte complexes are much higher than chitosan’s. Additionally, the authors observed that poly(acrylic acid)-chitosan complexes made from high molecular weight PAA showed a greater inhibition against *P. aeruginosa* and *P. oleovorans* than low molecular weight PAA [33]. In the present study it was noted that, along with the increasing mass ratio of the polymers, antimicrobial activity of sodium alginate-chitosan polyelectrolyte complex increases, which is in accordance with Ortega-Ortiz et al. results [36].

Chitosan is believed to chelate certain ions from the lipopolysaccharide (LPS) layer of the outer membrane of bacteria or to exhibit electrostatic interactions among its NH_3_+ groups and the negative charges of microbial cell membrane. In both cases, cell permeability increases releasing, key cellular components of bacteria [37,38]. In polyelectrolyte complexation carboxylic group of alginate interact with amino group of chitosan. After complexation, the number of un-bonded NH_3_+ groups (able to interact with negatively-charged cell membranes) presented in chitosan is lowered by interaction with the COO- of alginate. Taking into account mechanisms of the antimicrobial action of chitosan presented in the literature, its properties should be inhibited after complexing polyelectrolyte with polyanion. Different results of our study suggest that other factors could have an impact on PEC antibacterial behaviour against meat microbiota. Qi et al. and Ing et al. demonstrated that chitosan nanoparticles exhibited higher antimicrobial activity than chitosan solution due to their small particles and high surface charge [39,40]. SEM (scanning electron microscope) and MALDI-TOF (matrix-assisted laser desorption/ionization technique with time of flight analyser) analysis presented in our earlier studies confirmed creating sodium alginate-chitosan complex aggregates of various sizes dependent on the polymer ratios. PEC solutions consist of multiple polyelectrolyte complex particles suspended in excess of one of the polymers [15]. Those aggregates possess net charges different than zero and it is possible that they act like nanoparticles, which consequently exhibit greater antimicrobial properties than single chitosan solutions. This theory is also supported by the fact that alginate does not possess intrinsic antimicrobial features, so a synergistic antimicrobial effect is unlikely. According to Seon et al. the development of bacterial adhesion-resistant surfaces is possible by the use of hydrophilic polymers that can inhibit contact of bacteria to the surface due to the strong affinity of the polymers to water molecules [41]. Strong affinity to water of sodium alginate-chitosan complex films was confirmed in our earlier studies [25]. The greatest antimicrobial properties of PEC samples in connection with their high hydrophilicity may suggest that Seon et al. were correct [41]. The carboxylic group of alginate interacts with the amino group of chitosan resulting in numerous physical and chemical changes: reduced solubility in water, increase in viscosity due to sol-gel transition, and increased resistance to mechanical deformation [25]. All aforementioned changes are linked to the enhanced barrier properties of mixed polyelectrolytes, which may inhibit microbe growth due to decreased oxygen permeability. Summarizing, the limitation of microbial colonization on meat coated with a PEC surface could be the result of several factors: the nanoparticle-like character of the created complex aggregates, the bacterial adhesion-resistant surface of PEC, the barrier properties against oxygen, and the increased charge density due to polyelectrolyte complexation.

#### 2.2.3. Sensory Analysis

**Figure 3:**
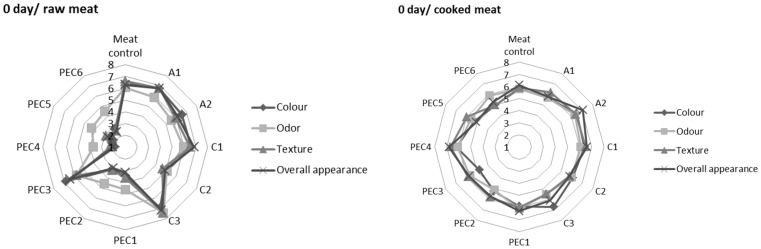

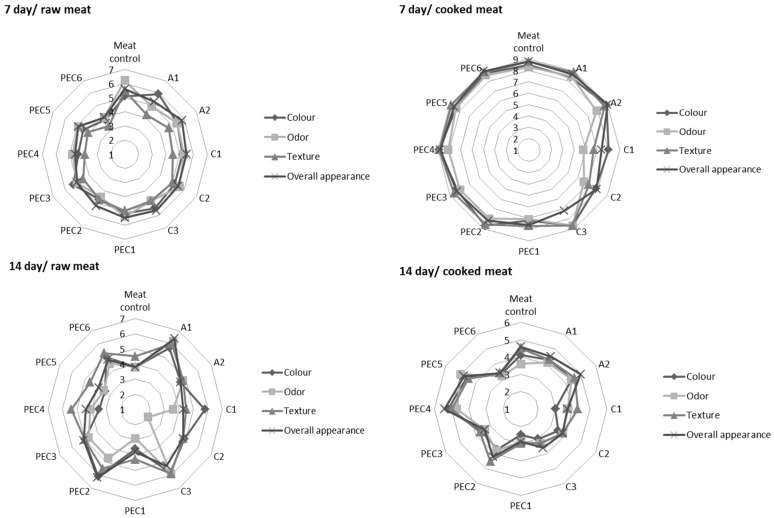
Sensory acceptance of raw (left column) and cooked (right column) pork meat uncovered/covered with sodium alginate (A1, A2), chitosan (C1, C2, C3), and sodium alginate-chitosan polyelectrolyte complex (PEC1–PEC6) during 0, 7, and 14 days of chill storage. A nine point hedonic scale was expressed as numbers 1–9 with boundary indications: ‘I do not like very much’ (1), ‘I neither like nor dislike’ (5), and ‘I like very much’ (9); *n* = 3.

Results of sensory analysis of raw and cooked pork meat uncovered/covered with A, C, and PEC were presented in Figure 3. Greater differentiation in the acceptance of the product was observed in the case of raw meat samples. At zero days of storage samples PEC4, PEC5, PEC6 were characterized by the lowest quality factors of all variants. These results can be associated with a less homogeneous structure and the milky colour of those hydrosols. Samples A1, A2, C1, C3, and PEC3 were perceived as fresh meat not covered by the experimental hydrosols. After seven days of storage, overall better acceptance of the experimental product was observed compared to day 0, which could be connected with the occurrence of merging process between hydrosol and meat tissue. After 14 days of storage, a decrease in sensory attributes of meat and meat-hydrosol samples was observed. Sample A1, C3, and PEC2 have been recognized as those of the best quality. Sensory panellists determined that samples C1, C2, and PEC1 were characterized by less appealing odour, which confirms unfavourable changes in meat tissue of these samples at 14 days of storage. Less visible differences in sensory analysis were observed in cooked samples. The acceptability of the product decreases with storage time but was more uniform across different variants. At day 0 no significant differences in sensory attributes of cooked samples were observed. After one week of storage only samples C1, C2, and PEC1 differed from the panellists’ standards. After two weeks of storage samples PEC4 and PEC5 were recognized as the best quality in comparison to cooked control and other cooked meat-hydrosol variants.

## 3. Materials and methods

### 3.1. Chemicals

Chitosan (molecular weight (Mw) ≈ 150 kDa, degree of deacetylation (DD) 82.84%, viscosity of 0.5% solution *w*/*v* in 1% solution. lactic acid: 0.03 ± 0.02 Pa∙s) was supplied by Sigma-Aldrich (St. Louis, MO, USA). Sodium alginate extracted from *Laminaria digitata* (viscosity of 0.5% solution 0.04 ± 0.01 Pa∙s, particle size maximum 5% > 400 μm, M:G ratio = 1.4) was supplied by Danisco (Copenhagen, Denmark). Glycerol was from Sigma-Aldrich (St. Louis, MO, USA) and 80% lactic acid was purchased from Purac (Gorinchem, The Netherlands).

### 3.2. Hydrosols Preparation

Polyelectrolyte complexes were produced from oppositely-charged polysaccharides: sodium alginate—A (polyanion), and chitosan—C (polycation). Sodium alginate powder (0.6% and 1.2%, *w*/*v*) was dissolved in deionized water by stirring (RW 20 digital, IKA^®^-Werke GmbH & Co. KG, Staufen, Germany) at 350 rpm for 24 h. Similarly, 1.0%, 1.5%, and 2.0% of chitosan solution (*w*/*v*) was prepared in 1% solution of lactic acid (*v*/*v*). To obtain a desirable (Table 3) concentration of pure polysaccharides (A1, A2, C1, C2, C3) 50 mL of base A and C solutions were diluted with 50 mL of deionized water. Polyelectrolyte complexes (PEC1–PEC6) were prepared by dropwise addition of (1 mL/s) 50 mL of base chitosan solution to 50 mL of base alginate solution during a constant homogenization process (IKA T18 basic, ULTRA TURRAX, IKA^®^-Werke GmbH & Co. KG). Glycerol (30% of dry weight of used polysaccharide) was used as a plasticizer and added to hydrosol solutions. The binding degree of the polyelectrolyte complex was adjusted by variable mutual content of A and C and presented as an A/C mutual mass ratio (Table 3). In the first part of the experiment A, C, and PEC hydrosols were investigated for DPPH radical scavenging activity and FRAP. Subsequently, A, C, and PEC were applied on raw meat to evaluate their effect on its total antioxidant capacity, microbiological, and sensory quality. Afterwards, raw meat samples coated with A, C, and PEC were cooked and tested for total antioxidant capacity and sensory quality.

### 3.3. First Part of the Experiment—The Analysis of Hydrosols

#### 3.3.1. DPPH Radical Scavenging Activity

Free radical scavenging activity of the pure polysaccharides (A, C) and sodium-alginate polyelectrolyte complex hydrosols (PEC) were determined by the method of Chen et al. [42]. Forty milligrams of 2,2-diphenyll- picrylhydrazyl was dissolved in 100 mL of ethanol. Then stock solution of DPPH was diluted with 100 mL of deionized water. The working solution of DPPH was prepared by diluting 200 mL stock DPPH solution with 800 mL of water/ethanol (50:50, *v*/*v*) mixture. The 0.5 mL of freshly prepared DPPH—ethanol solution was mixed with 1 mL of the tested sample and 1 mL of ethanol. The reaction mixture was shaken well and incubated at ambient temperature for 30 min. The reduction of the DPPH free radicals was measured by reading the absorbance at 517 nm. The antioxidant activity of samples was expressed as micromoles of Trolox per milliliter of the hydrosol (μM Trolox/mL).

#### 3.3.2. Ferric Reducing Antioxidant Power—FRAP

The ferric reducing antioxidant power assay (FRAP) was performed according to Benzie and Strain [43]. The working solution was prepared by mixing: 25 mL acetate buffer (300 mM); 2.5 mL TPTZ (10 mM 2,4,6-tripyridyls-triazine); 2.5 mL ferric chloride (20 mM FeCl_3_∙6H_2_O) solution. Experimental hydrosols (0.2 mL) were added to water (0.8 mL), mixed with the working FRAP solution (3 mL), and were incubated for 10 min in dark conditions. Reduction of a ferric tripyridyltriazine complex (Fe^3+^-TPTZ) to the ferrous form (Fe^2+^) was spectrophotometrically measured at 593 nm. Results were expressed as μM Fe^2+^/mL.

### 3.4. Second Part of the Experiment—The Application Analysis

#### 3.4.1. Meat Samples Preparation

The research material was raw pork muscle (*longissimus thoracis*) 48 h post-mortem originating from the Dworecki Meat Plant (Golejowo, Poland). Meat samples with 5 cm × 5 cm dimensions were immersed for 60 s in A, C, and PEC hydrosols, and then suspended for 5 min to drain the excess solution. Meat material was divided into two experimental groups: raw and cooked meat coated with experimental hydrosols. Raw meat-hydrosol samples and control meat (uncoated with hydrosol) were put into beakers and cooked for 10 min at 80 °C to achieve 72 °C at the geometric center of the sample. After cooking, samples were immediately cooled to 20 °C in an ice bath. Raw and cooked meat samples coated with hydrosol and uncoated meat controls (raw and cooked) were immediately packed in a modified atmosphere of 80% O_2_/20% CO_2_ (Air Products, Allentown, PA, USA) and stored under refrigeration (4 °C) for 0, 7, and 14 days.

#### 3.4.2. Total Antioxidant Capacity (TAC) Measurements

TAC of meat samples was determined by the QUENCHER method described by Serpen et al. [24]. After a certain time of storage, raw/cooked meat samples coated with A, C, or PEC and meat controls (raw/cooked) were homogenized and freeze dried (−40 °C) for 48 h. Freeze dried meat samples were ground to powder using a mortar. Ten milligrams of powdered meat sample was mixed with 10 mL of DPPH (1,1-diphenyl-2-picrylhydrazyl) or FRAP working solution. DPPH and FRAP working solutions were prepared as previously described. The mixture was shaken at 300–400 rpm at room temperature in the dark for 60 min (FRAP assay) and 120 min (DPPH assay). Then the mixture was centrifuged at 9200× *g* for 2 min. The absorbance of the clear supernatant (2 mL) was measured at 525 nm (DPPH assay) and 593 nm (FRAP assay). Results were expressed in mmol Trolox eq/kg of meat (d.w).

#### 3.4.3. Microbiological Analysis

Meat samples coated with 0.5% lactic acid solution (LA) and hydrosols (A, C, PEC) were prepared, packed and stored as previously described. After the predetermined period of storage packed samples were subjected to microbial analysis. Sterile cotton swabs were used for meat surface sampling in accordance with ISO 18593:2004 [44]. The template with the opening size of 5 cm × 4 cm was used to sampling from the same surface area each time. The swab was placed in a test tube with sterile 0.9% *w*/*v* saline solution (first dilution, 10^−1^), and further dilutions were prepared [45]. Determination of the total number of micro-organisms (TNM), was carried out using the plate method [46]. Colonies were counted after 72 h of incubation at 30 °C on culture medium. Psychrotrophs were incubated at 6 °C for 10 days according to ISO 17410:2001 [47]. A culture medium of yeast and molds was prepared according to ISO 21527-1:2008 [48] and plates were incubated for five days at 25 °C. The number of lactic acid bacteria was determined in accordance with ISO 13721:1995 [49] using MRS (de Man, Rogosa, and Sharpie) agar (Sigma-Aldrich). Colonies were counted after incubation at 30 °C for 72 h. Enumeration of Enterobacteriaceae was carried out by counting colonies cultured on the VRBG (violet red bile glucose agar, Sigma Aldrich) plates after 24 h incubation in 37 °C [50]. The CFU per milliliter of each sample was calculated as shown in the following equation:

(1) $$N=\frac{\sum^{\text{​}}C}{\left\{\left(n_{1}\times 1\right)+\;\left(n_{2}\times 0.1\right)\right\}\;d}$$

where N is the number of colonies per milliliter of the product (CFU/mL), ∑*C* is the counted sum of all colonies in all plates, *n*_1_ is the counted number of plates in lower dilution, *n*_2_ is the counted number of plates in higher dilution, and *d* is the dilution level corresponding to the first count (*n*_1_).

The results were expressed as log CFU per square cm of meat surface and calculated using the following formula:

(2) $$N_{s}=\frac{N\times F}{A}$$

where N is the number of colonies per milliliter of the product (CFU/mL), *F* is the amount of (mL) of dilution fluid, and *A* is the investigated surface (cm^2^).

#### 3.4.4. Sensory Analysis

Raw and cooked meat samples coated with hydrosols and uncoated control samples (raw/cooked) were MAP-packaged and refrigerated at 4 °C as previously described. Sensory evaluation was accomplished at day 0 and repeated after 7 and 14 days of storage. Samples were analysed for their colour, odour, texture and overall acceptability by 20 panellists familiar with pork meat evaluation. All samples were served in the clear Petri dishes. Sensory evaluation was carried out in individual booths under controlled conditions of light, temperature, and humidity. Sensory qualities of the samples were evaluated using a nine-point hedonic scale with boundary indications: ‘I do not like very much’ (1), ‘I neither like nor dislike’ (5), and ‘I like very much’ (9).

### 3.5. Statistical Analysis

Statistical analysis was performed with one-way (hydrosols properties) or two-way (application experiment) analysis of variance (ANOVA) using Statistica 10 (StatSoft, Krakow, Poland). Differences between mean values were identified by a Duncan test with a confidence level at *p* < 0.05. All experiments were performed in triplicate.

## 4. Conclusions

A polyelectrolyte complex made on the basis of sodium alginate and chitosan is a promising material that may be used to maintain the quality of meat due to its antimicrobial and antioxidant properties. PEC hydrosols were characterized by reduced free radical scavenging activities of DPPH in comparison to chitosan samples. On the other hand, samples PEC3 and PEC6 were characterized by the greatest ferric reducing antioxidant power of all variants. Polyelectrolyte coating of meat disturbs the contact with the external environment, thus inhibiting the negative influence of cooking and oxygen on meat tissue. Consequently, coating of meat with experimental hydrosols increased the TAC of raw/cooked meat and reduced the loss in TAC caused by thermal treatment of meat by 45%. Application of PEC significantly reduced the meat microbiota and extended shelf-life of raw pork by at least two weeks. Sodium alginate-chitosan complexes possess greater antimicrobial properties against TNM, YM, P, and LAB than A or C hydrosols. Sensory acceptance of raw/cooked control meat and meat-hydrosol samples gradually decreased during storage and depended on hydrosol composition. The relationship between the PEC’s binding degree and its biological characteristics was confirmed and investigation of this dependence should be continued in the future.

## Figures and Tables

**Table 1:** Total antioxidant capacity (TAC) of raw and cooked pork meat covered/uncovered with sodium alginate, chitosan, and sodium alginate-chitosan polyelectrolyte complex during 0, 7, and 14 days of chill storage.

Variant	Storage Time (Days)	DPPH		FRAP	
		Raw Meat	Cooked Meat	Raw Meat	Cooked Meat
Meat control	0	15.38 ± 0.14 ^hi^	15.06 ± 0.39 ^i−l^	11.97 ± 0.41 ^ab^	9.43 ± 0.19 ^bc^
A1		13.08 ± 0.18 ^a–c^	12.61 ± 0.22 ^a–c^	17.85 ± 0.64 ^e–h^	14.26 ± 0.15 ^k^
A2		14.77 ± 0.04 ^e–h^	13.74 ± 0.12 ^d–h^	18.06 ± 0.29 ^e–i^	16.14 ± 0.19 ^h^
C1		13.98 ± 0.08 ^c^^–^^f^	12.76 ± 0.11 ^a–d^	10.77 ± 0.31 ^a^	13.30 ± 0.18 ^ij^
C2		14.24 ± 0.01 ^d^^–^^g^	13.77 ± 0.22 ^d–h^	11.96 ± 0.13 ^ab^	12.62 ± 0.60 ^hi^
C3		15.01 ± 0.13 ^f^^–^^h^	14.55 ± 0.11 ^g–k^	16.61 ± 0.35 ^e^	13.54 ± 0.05 ^jk^
PEC1		13.04 ± 0.68 ^a^^–^^c^	12.98 ± 0.53 ^b–e^	11.63 ± 1.05 ^ab^	11.28 ± 0.17 ^fg^
PEC2		12.63 ± 1.01 ^ab^	12.33 ± 0.60 ^ab^	16.68 ± 0.45 ^e^	11.65 ± 0.24 ^g^
PEC3		16.22 ± 0.22 ^i^	13.58 ± 0.05 ^c–g^	14.60 ± 0.96 ^d^	14.17 ± 0.48 ^k^
PEC4		12.17 ± 0.35 ^a^	11.95 ± 0.21 ^a^	12.15 ± 0.40 ^ab^	12.70 ± 0.24 ^hi^
PEC5		12.80 ± 0.39 ^ab^	13.10 ± 0.22 ^b–f^	14.68 ± 1.62 ^d^	15.09 ± 0.45 ^l^
PEC6		14.17 ± 0.06 ^d^^–^^f^	13.89 ± 0.08 ^e–h^	14.38 ± 0.40 ^cd^	15.30 ± 0.18 ^lm^
Meat control	7	15.58 ± 0.42 ^hi^	17.58 ± 0.36 ^op^	13.09 ± 0.15 ^bc^	8.90 ± 0.15 ^b^
A1		15.27 ± 0.01 ^g–i^	15.28 ± 0.18 ^j–l^	17.42 ± 0.14 ^e–g^	20.14 ± 0.10 ^su^
A2		18.50 ± 0.24 ^k^	19.37 ± 0.16 ^rs^	19.30 ± 0.15 ^h–j^	21.38 ± 0.11 ^t^
C1		13.88 ± 0.03 ^c^^–^^e^	14.11 ± 0.09 ^f–i^	17.18 ± 0.12 ^ef^	9.84 ± 0.11 ^cd^
C2		14.54 ± 0.13 ^e^^–^^h^	14.71 ± 0.09 ^h–k^	17.79 ± 0.03 ^e–h^	10.68 ± 0.05 ^ef^
C3		15.53 ± 0.06 ^hi^	16.00 ± 0.26 ^lm^	19.85 ± 0.12 ^jk^	12.06 ± 0.30 ^gh^
PEC1		14.08 ± 0.04 ^c^^–^^f^	13.92 ± 0.12 ^e–h^	19.54 ± 0.09 ^ij^	11.79 ± 0.26 ^g^
PEC2		14.55 ± 0.59 ^e^^–^^h^	15.52 ± 0.26 ^kl^	21.74 ± 0.20 ^l^	19.07 ± 0.02 ^qr^
PEC3		16.21 ± 0.45 ^i^	16.69 ± 0.19 ^m–o^	21.61 ± 0.27 ^l^	20.98 ± 0.05 ^t^
PEC4		13.35 ± 0.28 ^b^^–^^d^	14.43 ± 0.13 ^g–j^	21.34 ± 0.05 ^kl^	16.43 ± 0.32 ^no^
PEC5		15.55 ± 0.66 ^hi^	14.79 ± 0.11 ^h–k^	21.28 ± 0.50 ^kl^	18.66 ± 0.24 ^q^
PEC6		17.31 ± 0.07 ^j^	16.50 ± 0.14 ^mn^	23.74 ± 0.36 ^mn^	21.57 ± 0.16 ^t^
Meat control	14	17.56 ± 0.43 ^j^	17.49 ± 0.27 ^op^	12.94 ± 0.13 ^bc^	7.90 ± 0.03 ^a^
A1		19.27 ± 0.06 ^kl^	14.97 ± 0.62 ^i–k^	24.11 ± 0.41 ^n^	20.98 ± 0.05 ^tu^
A2		21.24 ± 0.09 ^n^	19.77 ± 1.03 ^rs^	22.52 ± 0.43 ^lm^	21.19 ± 0.21 ^t^
C1		20.71 ± 0.07 ^mn^	19.00 ± 0.03 ^qr^	16.86 ± 0.33 ^ef^	9.35 ± 0.23 ^bc^
C2		22.57 ± 0.25 ^o^	20.28 ± 0.10 ^st^	12.95 ± 0.12 ^bc^	10.38 ± 0.45 ^de^
C3		24.06 ± 0.06 ^p^	21.05 ± 0.16 ^t^	18.36 ± 0.89 ^f–j^	15.95 ± 0.28 ^mn^
PEC1		18.86 ± 0.33 ^kl^	17.39 ± 0.13 ^n–p^	14.76 ± 0.69 ^d^	17.64 ± 0.82 ^p^
PEC2		19.78 ± 0.17 ^lm^	19.00 ± 0.24 ^qr^	17.16 ± 0.35 ^ef^	17.15 ± 0.36 ^op^
PEC3		20.38 ± 0.36 ^mn^	20.89 ± 0.26 ^t^	19.92 ± 0.08 ^jk^	21.22 ± 0.05 ^t^
PEC4		19.72 ± 0.09 ^lm^	13.77 ± 0.59 ^d–h^	18.80 ± 0.27 ^g–j^	19.65 ± 0.17 ^rs^
PEC5		20.99 ± 0.03 ^n^	18.35 ± 0.37 ^pq^	22.51 ± 0.54 ^lm^	20.93 ± 0.18 ^t^
PEC6		23.13 ± 0.42 ^op^	19.83 ± 0.03 ^rs^	24.22 ± 0.10 ^n^	21.69 ± 0.12 ^t^

Values with different letters (a–t) within the same column differ significantly (*p* < 0.05), ± standard error, results expressed as mmol Trolox eq/kg of meat, *n* = 3.

**Table 2:** The effect of sodium alginate (A1, A2), chitosan (C1, C2, C3), sodium alginate-chitosan complex (PEC1-PEC6), and 0.5% lactic acid solution (LA) application on surface microbiota of raw pork meat during 0, 7, and 14 days of chill storage.

Variant	Storage Time (Days)	TVC	YM	P	LAB	*Enterobacteriaceae*
LA	0	3.03 ± 0.03 ^e^	1.28 ± 0.03 ^b–e^	2.03 ± 0.04 ^b^	1.64 ± 0.03 ^c^	nd
Meat control		3.87 ± 0.02 ^i^	2.26 ± 0.02 ^i^	3.87 ± 0.02 ^g^	1.70 ± 0.01 ^d^	
A1		3.79 ± 0.01 ^i^	2.15 ± 0.02 ^hi^	3.63 ± 0.03 ^f^	nd	
A2		3.74 ± 0.01 ^hi^	2.04 ± 0.02 ^g–i^	3.59 ± 0.03 ^f^	nd	
C1		3.67 ± 0.00 ^h^	1.73 ± 0.09 ^f–h^	3.11 ± 0.05 ^e^	1.47 ± 0.02 ^b^	
C2		3.51 ± 0.00 ^g^	1.69 ± 0.08 ^e–g^	2.35 ± 0.01 ^c^	1.06 ± 0.05 ^a^	
C3		3.37 ± 0.02 ^f^	1.52 ± 0.02 ^d–f^	2.16 ± 0.05 ^b^	nd	
PEC1		2.98 ± 0.01 ^e^	1.07 ± 0.18 ^a–c^	2.34 ± 0.05 ^c^	nd	
PEC2		2.68 ± 0.11 ^d^	1.19 ± 0.02 ^a–e^	2.93 ± 0.06 ^d^	nd	
PEC3		2.36 ± 0.04 ^c^	1.04 ± 0.15 ^ab^	2.37 ± 0.12 ^c^	nd	
PEC4		2.70 ± 0.02 ^d^	0.80 ± 0.39 ^a^	1.64 ± 0.02 ^a^	nd	
PEC5		2.05 ± 0.04 ^b^	1.50 ± 0.02 ^c–f^	1.61 ± 0.03 ^a^	nd	
PEC6		1.93 ± 0.02 ^a^	1.38 ± 0.39 ^b–f^	2.95 ± 0.05 ^d^	nd	
LA	7	3.64 ± 0.02 ^f^	2.75 ± 0.04 ^f^	2.20 ± 0.05 ^c^	3.01 ± 0.09 ^f^	nd
Meat control		6.38 ± 0.08 ^k^	3.36 ± 0.06 ^h^	4.46 ± 0.02 ^i^	4.26 ± 0.01 ^h^	
A1		5.57 ± 0.01 ^i^	3.12 ± 0.06 ^g^	4.40 ± 0.04 ^i^	3.95 ± 0.04 ^g^	
A2		5.89 ± 0.06 ^j^	3.03 ± 0.01 ^g^	4.33 ± 0.06 ^i^	4.15 ± 0.04 ^h^	
C1		4.10 ± 0.03 ^h^	2.19 ± 0.01 ^e^	3.50 ± 0.02 ^h^	2.97 ± 0.01 ^f^	
C2		3.90 ± 0.01 ^g^	2.09 ± 0.05 ^de^	2.66 ± 0.04 ^f^	2.49 ± 0.00 ^e^	
C3		3.16 ± 0.00 ^e^	1.66 ± 0.02 ^b^	2.28 ± 0.06 ^cd^	2.10 ± 0.05 ^d^	
PEC1		3.13 ± 0.04 ^e^	1.89 ± 0.05 ^c^	2.49 ± 0.01 ^e^	1.46 ± 0.10 ^b^	
PEC2		2.92 ± 0.01 ^d^	1.51 ± 0.10 ^a^	2.91 ± 0.04 ^g^	1.26 ± 0.15 ^a^	
PEC3		2.55 ± 0.01 ^b^	1.95 ± 0.04 ^cd^	2.69 ± 0.10 ^f^	1.76 ± 0.03 ^c^	
PEC4		2.72 ± 0.00 ^c^	1.50 ± 0.02 ^a^	1.72 ± 0.05 ^a^	1.62 ± 0.00 ^bc^	
PEC5		2.21 ± 0.05 ^a^	2.03 ± 0.04 ^cd^	1.93 ± 0.05 ^b^	1.72 ± 0.01 ^c^	
PEC6		2.19 ± 0.01 ^a^	1.92 ± 0.07 ^c^	2.38 ± 0.02 ^de^	2.36 ± 0.03 ^e^	
LA	14	4.93 ± 0.07 ^e^	4.08 ± 0.03 ^e^	2.61 ± 0.05 ^cd^	4.90 ± 0.01 ^j^	nd
Meat control		7.86 ± 0.03 ^i^	4.21 ± 0.04 ^e^	6.31 ± 0.02 ^h^	6.18 ± 0.02 ^m^	
A1		7.74 ± 0.01 ^h^	4.14 ± 0.02 ^e^	5.56 ± 0.04 ^g^	5.47 ± 0.02 ^k^	
A2		7.70 ± 0.03 ^h^	4.13 ± 0.02 ^e^	5.49 ± 0.04 ^g^	5.51 ± 0.00 ^l^	
C1		5.91 ± 0.00 ^g^	3.43 ± 0.19 ^d^	3.82 ± 0.05 ^f^	3.70 ± 0.01 ^i^	
C2		5.54 ± 0.02 ^f^	3.23 ± 0.05 ^c^	2.79 ± 0.06 ^d^	3.29 ± 0.05 ^h^	
C3		3.82 ± 0.09 ^c^	2.65 ± 0.05 ^b^	2.56 ± 0.02 ^bc^	3.15 ± 0.01 ^g^	
PEC1		3.96 ± 0.00 ^d^	2.66 ± 0.03 ^b^	2.56 ± 0.00 ^bc^	2.28 ± 0.01 ^c^	
PEC2		2.91 ± 0.01 ^b^	2.16 ± 0.03 ^a^	3.06 ± 0.20 ^e^	2.17 ± 0.00 ^b^	
PEC3		2.90 ± 0.03 ^b^	2.21 ± 0.06 ^a^	3.25 ± 0.07 ^e^	2.56 ± 0.00 ^d^	
PEC4		2.95 ± 0.01 ^b^	2.31 ± 0.02 ^a^	1.97 ± 0.03 ^a^	2.60 ± 0.00 ^e^	
PEC5		2.62 ± 0.02 ^a^	2.33 ± 0.01 ^a^	2.07 ± 0.02 ^a^	2.05 ± 0.01 ^a^	
PEC6		2.65 ± 0.01 ^a^	2.16 ± 0.06 ^a^	2.38 ± 0.01 ^b^	2.69 ± 0.01 ^f^	

nd—colonies not detected, values with different letters (a–k) within the same column differ significantly (*p* < 0.05), ± standard error. TNM—total number of micro-organisms, YM—yeasts and moulds, P—psychrotrophic microorganisms, LAB—lactic acid bacteria, results expressed as log CFU/cm^2^, *n* = 3.

**Table 3:** Experimental design showing samples compositions and coding.

Coding		Polymers		A/C Mutual Mass Ratio
		A (%)	C (%)	
Controls	A1	0.30	0	-
	A2	0.60	0	
	C1	0	0.50	
	C2	0	0.75	
	C3	0	1.00	
Polyelectrolyte complex	PEC1	0.30	0.50	0.60
	PEC2	0.30	0.75	0.40
	PEC3	0.30	1.00	0.30
	PEC4	0.60	0.50	1.20
	PEC5	0.60	0.75	0.80
	PEC6	0.60	1.00	0.60

## Abbreviations

The following abbreviations are used in this manuscript:

A	Sodium alginate
C	Chitosan
CFU	Colonies-forming unit
DPPH	2,2-Diphenyll-picrylhydrazyl
E	Enterobacteriaceae
FRAP	Ferric reducing antioxidant power
HPMC	Hydroxypropyl methyl cellulose
LA	Lactic acid
LAB	lactic acid bacteria
LPS	Lipopolysaccharide
MAP	Modified atmosphere packaging
MRPs	Maillard Reaction Products
MRS	de Man, Rogosa and Sharpe agar
P	Psychrotrophs
PAA	Poly(acrylic acid)
PEC	Polyelectrolyte complex
SEM	Scanning electron microscope
TAC	Total antioxidant capacity
TBARS	Thiobarbituric acid reactive substances
TNM	Total number of micro-organisms
TPTZ	2,4,6-Tripyridyls-triazine
VRBG	Violet red bile glucose agar
YM	Yeast and molds
